# A phase 2 study of intraperitoneal carboplatin plus intravenous dose-dense paclitaxel in front-line treatment of suboptimal residual ovarian cancer

**DOI:** 10.1038/s41416-020-0734-9

**Published:** 2020-01-31

**Authors:** Kosei Hasegawa, Muneaki Shimada, Satoshi Takeuchi, Hiroyuki Fujiwara, Yuichi Imai, Norihiro Iwasa, Satoru Wada, Hidetaka Eguchi, Tetsuro Oishi, Toru Sugiyama, Mitsuaki Suzuki, Masahiko Nishiyama, Keiichi Fujiwara

**Affiliations:** 1 Department of Gynecologic Oncology, Saitama Medical University International Medical Center, Hidaka, Saitama 350-1298 Japan; 2 Project Research Division, Research Center for Genomic Medicine, Saitama Medical University, Hidaka, Saitama 350-1241 Japan; 3 Department of Obstetrics and Gynecology, Tottori University School of Medicine, Yonago, Tottori 683-8504 Japan; 4 Department of Obstetrics and Gynecology, Iwate Medical University, Morioka, Iwate 020-8505 Japan; 5 Department of Obstetrics and Gynecology, Jichi Medical University, Shimotsuke, Tochigi 329-0498 Japan; 6 Division of Translational Research, Research Center for Genomic Medicine, Saitama Medical University, Hidaka, Saitama 350-1241 Japan; 7 Department of Molecular Pharmacology and Oncology, Gunma University Graduate School of Medicine, Maebashi, Gunma 371-8511 Japan

**Keywords:** Ovarian cancer, Ovarian cancer

## Abstract

**Background:**

We evaluated the efficacy of intraperitoneal (IP) carboplatin in combination with dose-dense paclitaxel (ddTCip) for suboptimal residual ovarian cancer.

**Methods:**

This was a phase 2 study to evaluate ddTCip. Patients with stage II–IV ovarian carcinoma, who underwent primary cytoreductive surgery and had radiologically evaluable disease after surgery, were eligible to participate in this study. IP carboplatin (AUC = 6) was administered on day 1, and intravenous paclitaxel (80 mg/m^2^) was administered on days 1, 8 and 15. The primary endpoint was response rate. Secondary endpoints included progression-free survival (PFS), overall survival (OS) and safety. Interval- debulking surgery followed by the same regimen was allowed when indicated.

**Results:**

A total of 117 patients were considered eligible for this study prior to surgery and temporarily registered. Of the 117 patients, 76 patients met the inclusion criteria and were enrolled in this study. Fifty-nine (83.1%) patients had objective clinical responses. Median PFS and OS were 18.3 and 55.5 months, respectively. Sixty-four (84.2%) patients had grade 3/4 neutropenia, 43 (56.5%) patients had anaemia and 17 (22.4%) patients had thrombocytopenia. Port-related adverse events occurred in nine (11.8%) patients.

**Conclusions:**

Front-line chemotherapy with ddTCip therapy appears safe and effective, even for patients with suboptimal residual ovarian cancer.

**Trial registration:**

UMIN Clinical Trials Registry (ID: UMIN000001713) on February 16th, 2009.

## Background

There are two important chemotherapy approaches that achieved improved overall survival (OS) in patients with epithelial ovarian cancer (EOC) and are receiving increased attention in this area. One is intraperitoneal (IP) chemotherapy.^1^ The other is a combination chemotherapy consisting of dose-dense paclitaxel plus carboplatin (ddTC)^2^. Prior to the advent of bevacizumab and dose-dense paclitaxel, there were three large trials that included IP cisplatin. GOG104, 114, and 172 exhibited significantly improved OS within the IP arms, compared with the control arms.^1^ A meta-analysis showed that the hazard ratio (HR) was around 0.78 at the time of the NCI clinical announcement in 2006, and an updated meta-analysis reported a HR of 0.81 in 2014.^3^ Although IP chemotherapy plus cisplatin demonstrated better survival advantages for those with optimally debulked stage III ovarian cancer, it is not widely accepted as a standard chemotherapy. In fact, less than half of eligible women treated at NCI Comprehensive Cancer centers received this treatment, as reported by Wright et al.^4^ This is due to its association with potential toxicity and port-related events; in addition, IP chemotherapy has never been compared with standard TC treatment. IP administration of carboplatin, instead of cisplatin, might circumvent IP cisplatin-related toxicities.

The administration of IP therapy to patients with suboptimal disease is challenging. The original rationale for IP chemotherapy is that this route of administration produces a higher IP concentration of the drug, enabling longer exposure within the peritoneal cavity.^5^ The IP route is considered an enhanced local therapy. Consequently, most trials have been conducted in patients with optimally debulked ovarian cancer. However, in GOG104, larger OS hazard reductions were observed in larger residual tumours (1–2 cm), compared with smaller residual tumours (<1 cm) in the IP chemotherapy group.^5^ In addition, IP administration of carboplatin exhibits a preferable pharmacokinetic profile, compared with IV carboplatin. Miyagi et al. conducted a pharmacokinetic analysis to compare IP with IV carboplatin, and found that the platinum area under the curve (AUC) in the peritoneal cavity of patients who received IP carboplatin was 17 times higher than that observed in patients who received IV carboplatin. Nevertheless, the serum platinum AUC remained the same with both routes, suggesting that IP carboplatin is feasible, not only as a regional therapy, but also as a more reasonable route for systemic chemotherapy^6^. However, IP drug levels might not reflect drug delivery to tumour directly, and direct drug penetration from IP to tumour is limited by a number of factors, including fibrosis, adhesions, loculations and increased interstitial pressure.^7^

Dose-dense weekly paclitaxel and carboplatin (ddTC) therapy improves efficacy and OS in patients with ovarian cancer. A Japanese Gynecologic Oncology Group (JGOG) 3016 phase 3 clinical study demonstrated the superiority of ddTC, compared with standard TC.^2^ They observed prolonged progression-free survival (PFS) and long-term OS in patients treated with the ddTC, compared with those who received the conventional 3-week administration of TC.^2,8^ On the other hand, MITO7, GOG262 and most recently, ICON8, did not show any survival benefit in patients treated with ddTC compared with those treated with standard TC.^9–11^ In addition to differences in treatment regimens that could have an impact on clinical outcomes, there might be racial/ethnic differences in the responses to the ddTC therapy.

IP carboplatin may exhibit reduced toxicity, compared with IP cisplatin. IP carboplatin shows reasonable pharmacokinetics, even in cases of suboptimal disease. In addition, ddTC shows better efficacy than conventional TC. However, we have not prospectively evaluated the efficacy and the safety of ddTCip therapy for patients with advanced ovarian cancer, particularly in those with suboptimal disease.

## Methods

### Study design

This was a single-arm, open-label, multicentre phase 2 study in Japan. The protocol was registered within the UMIN Clinical Trials Registry (ID: UMIN000001713) on Feb 16th, 2009. The primary endpoint was response rate according to Response Evaluation Criteria in Solid Tumors (RECIST version 1.0). Secondary endpoints were PFS, OS and safety.

### Patients

Patients older than age 20, with FIGO stage II–V histologically confirmed EOC or primary peritoneal cancer, were enrolled. All patients had radiologically evaluable disease by RECIST after initial debulking surgery, with ECOG Performance Status scores of 0–2, and had not undergone any chemotherapy or radiation therapy. In addition, all patients should have an adequate organ function. All patients enrolled in this study were non-Hispanic Asians of Japanese descent. Patients were temporarily registered before surgery. The patients underwent primary surgery, and if the residual tumour was judged to be measurable by the surgeon, IP port was placed during the operation. If they met the inclusion criteria before front-line chemotherapy, they were enrolled in this study. They were recruited for a prospective phase 2 study of weekly IV administration of paclitaxel and 3-week IP bolus infusion of carboplatin [Development Organization for Frontier Medical Therapeutics (DOFMET) protocol #4], from March 2009 to March 2012. This study was approved by the local ethics committee of each participating institution, and all patients gave written informed consent.

### Chemotherapy

All the patients received IP bolus infusion of carboplatin (AUC = 6 on day 1 every 3 weeks) and IV administration of dose-dense paclitaxel (80 mg/m^2^, on days 1, 8 and 15, every 3 weeks). We defined more than six cycles of chemotherapy as the complete protocol treatment. Adverse effects were determined using National Cancer Institute Common Terminology Criteria for Adverse Effects (CTCAE-NCI), version 3.0.

Dose reductions were allowed depending on predefined levels of haematologic or non-haematologic toxicities (Supplementary Table 1).

### Surgery

If patients responded to the protocol treatment, interval-debulking surgery followed by the same regimen was allowed when indicated.

### Tumour assessment

Treatment response was assessed using the RECIST, version 1.0. After primary surgery and before the start of the chemotherapy, a chest-to-pelvis CT scan was performed. The scans were repeated after each cycle of chemotherapy until disease progression or the follow-up visit. CA125 measurements were required every 3 months, and CT scans were required at least every 6 months, or whenever indicated, during follow-up until progression.

### Statistical analysis

The sample size was calculated by the following hypothesis. When the expected response rate of the study treatment (IP carboplatin administration) is set to 75% compared with 60% of the response rate for the standard treatment (IV carboplatin administration), the sample size required for the superiority test by the one-sided test with α error = 0.05 and β error = 0.2 was 65.9. Considering dropout cases, the target accrual of cases was set to 80 cases.

We analysed toxicities in 76 patients who received at least one dose of chemotherapy. Efficacy analyses were performed on patients who completed more than one cycle of protocol chemotherapy (n = 71). PFS was defined as the time interval between registration and progression or death, whichever occurred first, or the last follow-up for patients alive without progression. OS was defined as the time interval between registration and death, or the last follow-up for patients alive. PFS and OS curves were estimated using the Kaplan–Meier method. All statistical analyses were conducted using statistical software JMP 10 (SAS Institute, Cary, NC, USA).

## Results

### Patient characteristics

Between March 2009 and March 2012, a total of 117 patients with EOC or primary peritoneal cancer, FIGO stage II–IV, who were considered eligible for this study, were temporarily registered prior to primary surgery. Of the 117 patients, 76 met the inclusion criteria and were enrolled. The major reasons for study disqualification were no measurable residual tumour after surgery, stage I disease, other malignancies (Krukenberg’s tumour, sarcoma and fallopian tube cancer) and PS3 or worse (see Supplementary Fig. 1). Baseline characteristics (*n* = 76) are shown in Table 1. The median follow-up was 37.8 months for surviving patients.

**Table 1:** Patient characteristics and compliance with chemotherapy (*n* = 76).

Factors	*N* (%)
Age	
Median (range)	62 (37–78)
FIGO stage	
II	1 (1.3)
III	46 (60.5)
IV	29 (38.2)
ECOG performance status	
0	58 (76.3)
1	11 (14.5)
2	7 (9.2)
Primary site	
Ovary	74 (97.4)
Peritoneum	2 (2.6)
Histological type	
High-grade serous	58 (76.3)
Endometrioid	3 (3.9)
Clear cell	3 (3.9)
Mucinous	2 (2.6)
Others	10 (13.2)
Interval-debulking surgery	
Yes	46 (60.5)
No	30 (39.5)
Residual disease (cm)	
1–2	2 (2.6)
2–5	24 (31.6)
>5	50 (65.8)
No. of cycles administered	
0	5 (6.6)
1	3 (3.9)
2	3 (3.9)
3	6 (7.9)
4	5 (6.6)
5	8 (10.5)
6 or more	46 (60.5)
Cause of treatment interruption	
Treatment completion	46 (60.5)
Toxicity	17 (22.4)
Progression	5 (6.6)
Refusal/others	8 (10.5)
Cycle delayed because of toxicity	
First cycle	12/76 (15.8)
Second cycle	32/70 (45.7)
Third cycle	22/66 (33.3)
Fourth cycle	26/63 (41.3)
Fifth cycle	29/57 (50.9)
Sixth or more cycles	22/48 (45.8)

### Treatment exposure

Table 1 also summarises treatment exposure and treatment interruption causes. Of the 76 patients, 46 (60.5%) completed more than six cycles, and they received a median of six protocol chemotherapy cycles. Most patients experienced delays, interruptions or dose modifications of at least one chemotherapy infusion because of adverse events. Fifty-seven (75.0%) and 35 (46.1%) patients required schedule delay and dose reduction due to the chemo-related toxicity, respectively.

The most common reason for discontinuation was completion of the planned treatment course in 46 patients (60.5%). Seventeen (22.4%) patients discontinued because of toxicity, and five (6.6%) patients were discontinued secondary to disease progression. The adverse events most commonly leading to the protocol discontinuation were haematological toxicities (*n* = 7, 9.2%), port-related complications (*n* = 6, 7.9%) and thrombosis (*n* = 2, 2.6%).

### Efficacy

Of the 76 patients enrolled, 71 (93.4%) patients completed at least one cycle of protocol treatment, and were subjected to efficacy analysis. Of the 71 patients, two patients (2.8%) were not evaluable because of absent tumour response confirmation via radiological images. The overall response rate by RECIST in the 71 patients was 83.1% (95% CI: 72.7–90.1). The confirmed complete response and partial response rates were 12.7% (95% CI: 6.8–22.4) and 70.4% (95% CI: 59.0–79.8), respectively (Table 2). The median duration of response in the 59 responding patients was 16.3 months (95% CI: 14.8–18.2). Interval-debulking surgery (IDS) followed by the same protocol regimen was allowed when indicated in this study. Forty-five (63.4%) patients were treated with IDS after two to six cycles of chemotherapy. Most of the responding patients clinically have no tumours at the end of the courses. They were observed just after finishing therapy, as long as there were no clinically evident diseases.

**Table 2:** Objective responses (*n* = 71).

	*N* (%)	95% CI
Complete response	9 (12.7)	6.8–22.4
Partial response	50 (70.4)	59.0–79.8
Stable disease	10 (14.1)	7.8–24.0
Progressive disease	0 (0)	
Not evaluable	2 (2.8)	
Objective response rate	59 (83.1)	72.7–90.1

**Fig. 1:**
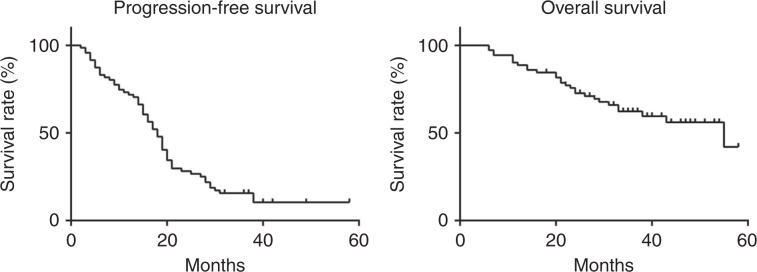
Kaplan–Meier curves of progression-free survival and overall survival.

At the time of data cut-off for this analysis (March 31, 2014: 24 months after enrolment of the last patient), PFS events were observed in 60 (84.5%) of the 71 patients. OS data were still relatively immature. We observed 28 deaths (39.4%) at the time of data cut-off. The median PFS was 18.3 months (95% CI, 15.5–20.2), and the median OS was 55.5 months (95% CI, 33.7–∞), at the time of data cut-off. The Kaplan–Meier estimates of PFS and OS are shown in Fig. 1.

### Safety

All enrolled patients received at least one dose of chemotherapy; thus, 76 patients were eligible for the toxicity analyses. The most severe toxicities are presented in Table 3. Grade 3/4 adverse events were reported in 75 patients (98.7%). There were no treatment-related deaths. The most common (grade 3/4) adverse events were haematological toxicities. Sixty-four (84.2%) patients had grade 3/4 neutropenia, 43 (56.5%) patients had anaemia and 17 (22.4%) patients had thrombocytopenia. Although grade 3 peripheral neuropathy was observed in only eight patients (10.5%), 27 (35.5%) patients experienced grade 2 peripheral neuropathy. Port-related adverse events occurred in nine (11.8%) patients, including infection (*n* = 5), pain (*n* = 2) and obstruction (*n* = 2).

**Table 3:** Adverse events (*n* = 76).

Adverse events	Grade 2	Grade 3	Grade 4
Leukocytopenia	24 (31.6%)	44 (57.9%)	4 (5.3%)
Neutropenia	8 (10.5%)	37 (48.7%)	27 (35.5%)
Anaemia	24 (31.6%)	35 (46.1%)	8 (10.5%)
Thrombocytopenia	13 (17.1%)	12 (15.8%)	5 (6.6%)
Fatigue	8 (10.5%)	1 (1.3%)	–
Anorexia	11 (14.5%)	2 (2.6%)	–
Nausea	21 (27.6%)	2 (2.6%)	–
Vomiting	13 (17.1%)	-	–
Constipation	26 (34.2%)	2 (2.6%)	–
Diarrhoea	5 (6.6%)	-	–
Neuropathy (sensory)	15 (19.7%)	6 (7.9%)	–
Neuropathy (motor)	12 (15.8%)	2 (2.6%)	–
Arthralgia	4 (5.3%)	1 (1.3%)	–
Myalgia	4 (5.3%)	1 (1.3%)	–
Alopecia	46 (60.5%)	-	–
Thrombosis	–	1 (1.3%)	1 (1.3%)
Port-related adverse events	–	9 (11.8%)	–

## Discussion

In this study, the objective response rate (ORR) for ddTCip was 83.1%. The previous two weekly paclitaxel trials: the ORRs were 56.0% for JGOG3016 and 56.2% for MITO7.^2,10^ As for patients’ outcomes, the median PFS and OS in this study were 18.3 and 55.5 months, respectively. PFS and OS for patients with suboptimal disease in the JGOG3016 trial were 17.6 and 51.2 months.^8^ A recent GOG262 trial to compare dose-dense weekly paclitaxel with 3-week paclitaxel in patients with unresected stage III/IV ovarian cancer revealed a median PFS and OS of 14.7 and 40.2 months in the weekly paclitaxel arm, respectively.^9^ These may be attributed to potential patient selection bias. Our phase 2 study had very specific eligibility criteria, and Phase 3 trials included a broader group of patients. We need a direct comparison between IP versus IV administration route for suboptimal residual disease. An increased survival was observed in EOC patients with decreased BRCA1 expression receiving IP chemotherapy.^12^ However, the study utilised immunohistochemistry for BRCA1 status that does not necessarily correlate with BRCA function or homologous recombination deficiency status. A recent report described that IP chemotherapy was associated with an improvement in PFS and OS in patients who had pathogenic *BRCA* mutations compared with the patients who did not.^13^ Both studies were retrospective analyses that have not been validated prospectively. The relationship between clinical outcomes and *BRCA* mutations requires further analysis. BRCA1 expression or *BRCA* mutation data were not available in this trial at the moment. However, the pharmacogenomic and gene expression array analyses for this trial are ongoing. The results may reveal possible future applications for precision medicine.

The overall toxicity profile in this study was like that of JGOG3016, except port-related adverse events. However, we observed a slightly lower rate of haematological toxicities in this trial, compared with JGOG3016. The incidence of grade 3/4 neutropenia (84.2%), anaemia (56.6%) and thrombocytopenia (22.4%) in this trial was lower than percentages observed in the dose-dense regimen group of JGOG3016.^2^ One reason might be pharmacokinetic differences that exist between IP and IV carboplatin administration. Nine (11.8%) patients had grade 3 or higher port-related adverse events. For the most part, this rate agrees with rates observed during previous IP trials. The proportion of patients who received six or more cycles of treatment in this study (60.5 %) was higher than that observed in the IP chemotherapy group of GOG172 (42%),^14^ but was equivalent to that observed in the dose-dense paclitaxel-treated group of JGOG3016 (62%). Therefore, compliance rates for ddTCip therapy will likely be superior to those of IP cisplatin therapy.

We need to validate if ddTCip is better than IV carboplatin plus dose-dense paclitaxel. A randomised P3 study is needed to prove this. The recent report from phase 3 GOG252 trial demonstrated that weekly paclitaxel plus IP carboplatin did not improve PFS compared with that of weekly paclitaxel plus IV carboplatin, and nearly mature OS data also demonstrate no difference in outcomes. There is no statistical expectation that a difference will emerge limited to mature OS with these data.^15^

A Canadian phase 2 trial of OV21 showed decreased rates of disease progression at 9 months post chemotherapy for patients treated with IP carboplatin and paclitaxel after optimal resection at the time of IDS. Unfortunately, this trial did not result in a P3 trial.^16^ Different outcomes were seen between Japanese (JGOG3016) and Caucasian (ICON8) patients with regard to dose-dense paclitaxel therapy, which was demonstrated to be more beneficial than standard 3-week paclitaxel therapy in Japanese population.^2,11^ Therefore, dose-dense paclitaxel plus IP carboplatin should be compared with dose-dense paclitaxel plus IV carboplatin, particularly in Japanese patients. The iPocc trial (GOTIC-001/JGOG3019; NCT01506856) comparing dose-dense paclitaxel plus IV with IP carboplatin is ongoing.^17^ It has just completed its enrolment in August 2016.

In conclusion, ddTCip chemotherapy shows remarkable efficacy in the treatment of patients with ovarian cancer who had suboptimal residual disease at the time of primary surgery. Except the incidence of port-related adverse events, ddTCip had a similar toxicity profile to that of the dose-dense arm of JGOG3016. The results of the ongoing iPocc trial would provide some important clues to resolving the remaining questions that surround IP carboplatin therapy.

## Supplementary information

Figure legends

Supplementary Figure1

Supplementary table 1

## Data Availability

Data are available through the Development Organization for Frontier Medical Therapeutics (DOFMET) in Japan.
