# Supplementary material for: A phase 2 study of intraperitoneal carboplatin plus intravenous dose-dense paclitaxel in front-line treatment of suboptimal residual ovarian cancer
Source: Br J Cancer. 2020 Jan 31;122(6):766–70. doi: 10.1038/s41416-020-0734-9 (PMC7078205; doi:10.1038/s41416-020-0734-9)
Supplement: Supplementary file 1 — Figure legends [file 41416_2020_734_MOESM1_ESM.docx]

**Figure legends**

**Figure 1. (A)** Kaplan–Meier estimates of progression-free survival and (**B)** overall survival curves (n = 71).
