# Supplementary figures and images for: A phase 2 study of intraperitoneal carboplatin plus intravenous dose-dense paclitaxel in front-line treatment of suboptimal residual ovarian cancer
Source: Br J Cancer. 2020 Jan 31;122(6):766–70. doi: 10.1038/s41416-020-0734-9 (PMC7078205; doi:10.1038/s41416-020-0734-9)

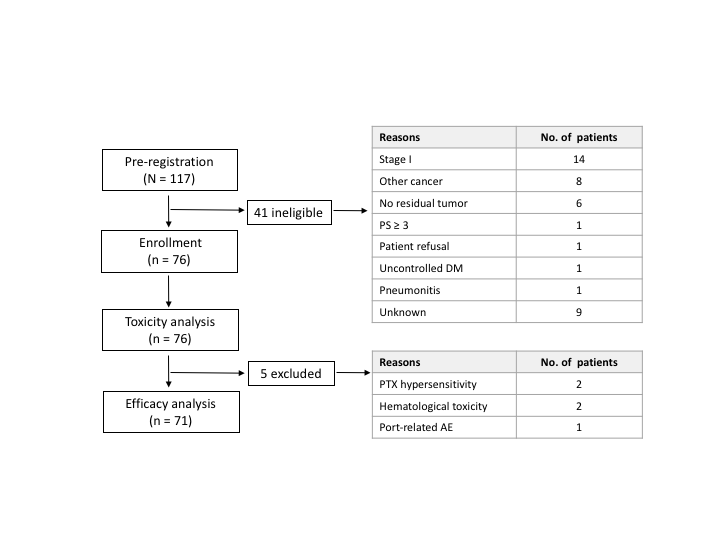

Supplement: Supplementary file 2 — Supplementary Figure1 [file 41416_2020_734_MOESM2_ESM.tif]
